# Supplementary material for: GigaSOM.jl: High-performance clustering and visualization of huge cytometry datasets
Source: Gigascience. 2020 Nov 18;9(11):giaa127. doi: 10.1093/gigascience/giaa127 (PMC7672468; doi:10.1093/gigascience/giaa127)
Supplement: giaa127_Response_to_Reviewer_Comments_Original_Submission [file giaa127_response_to_reviewer_comments_original_submission.pdf]

Author's Response To Reviewer Comments

Close

We are grateful to both reviewers for their comments and motivating remarks about the manuscript and the software.

The updated manuscript version contains minor stylistic corrections that we noticed during the editing process, a fixed reference to FlowSOM, and a minor change in section "Methods", which was requested by Reviewer #2 in this comment:

> One area of improvement is to better clarify the methodological developments
> presented here vs. those already adopted by the field. This has already been
> done to a good degree but could be made more explicit, particularly with
> regards to Batch Training of SOMs, Map Reduce, etc.

We originally aimed to discuss the utility of the new tool for immediate (and relatively straightforward) application to existing data, which resulted in this clarification being omitted in the first version of the manuscript.

After contemplating about this comment, we now see that this was also partly caused by the fact that the main methodological development in GigaSOM is particularly subtle: The software brings together the software engineering (to produce a software piece that solves the newly appearing problem) and a counter-acting "limit" on the implementation complexity (to make sure that the software is accessible to users and flexible for customization and future improvement, despite throwing away possible complicated optimizations and the benefits of using the popular Python and R environments). We believe that we have clarified this motivation and the main benefits of the design choices, and referenced a sufficient spectrum of possible alternative approaches in the "Methods" section, which is now updated with the new subsection "Implementation methodology".

Additionally, the editorial office requested the following improvement:

> In addition, please register any new software application in the bio.tools
> and SciCrunch.org databases to receive RRID (Research Resource Identification
> Initiative ID) and biotoolsID identifiers, and include these in your
> manuscript. This will facilitate tracking, reproducibility and re-use of your
> tool.

GigaSOM is now registered in both bio.tools and SciCrunch, and the IDs (biotoolsID and RRID) are reported in the manuscript backmatter. We also added an explicit name of the package as registered in the official Julia packaging system, which we believe to be quite useful, as it is the arguably simplest way of installing GigaSOM.

Close
