# Supplementary material for: GigaSOM.jl: High-performance clustering and visualization of huge cytometry datasets
Source: Gigascience. 2020 Nov 18;9(11):giaa127. doi: 10.1093/gigascience/giaa127 (PMC7672468; doi:10.1093/gigascience/giaa127)
Supplement: giaa127_Response_to_Reviewer_Comments_Revision_1 [file giaa127_response_to_reviewer_comments_revision_1.pdf]

Author's Response To Reviewer Comments

Close

In this revision, we respond mainly to the specific requirements raised by the assigned handling editor.

The revision adds RRIDs of the referenced software tools (added where available), author ORCIDs, and the added link to GigaDB data in the backmatter. Together with the RRID of R language, we also added the citation to the language description, for completeness.

The manuscript is otherwise without any significant changes.

Close
