# Supplementary material for: GigaSOM.jl: High-performance clustering and visualization of huge cytometry datasets
Source: Gigascience. 2020 Nov 18;9(11):giaa127. doi: 10.1093/gigascience/giaa127 (PMC7672468; doi:10.1093/gigascience/giaa127)
Supplement: giaa127_Reviewer_1_Report_Original_Submission [file giaa127_reviewer_1_report_original_submission.pdf]

Reviewer Report

Title: GigaSOM.jl: High-performance clustering and visualization of huge cytometry datasets

Version: Original Submission **Date:** 8/14/2020

Reviewer name: Chris Armit

Reviewer Comments to Author:

This technical note describes GigaSOM.jl, a Julia toolkit for clustering and visualisation of large cytometry data. The core objective achieved by GigaSOM.jl is the ability to quickly cluster and visualise large flow cytometry datasets through use of Self-Organising-Maps (SOM), which enable efficient and accurate clustering of millions of cells. Validation of GigaSOM.jl clustering was established by comparison with FlowSOM, which is an established toolkit that has been described previously (Gassen et al., FlowSOM: Using selforganizing maps for visualization and interpretation of cytometry data. Cytometry Part A 2015;87(7):636-645.). From a 'Big Data' perspective, the utility of the GigaSOM.jl toolkit is showcased in the context of a large flow cytometry dataset that was generated by the International Mouse Phenotyping Consortium (IMPC) and that consisted of 1,167,129,317 splenocytes from 2,905 samples, including genetically modified single-gene knockout mice and control samples. Asinh transformations (i.e. inverse hyperbolic sine transformations) of fluorescent marker expression values from the splenocyte dataset were used to train a 32Ã—32 SOM, and this was subsequently rasterized to produce the visualisation shown in Figure 5. The manuscript is well written and I see great reuse potential in the GigaSOM.jl toolkit for clustering and dimension reduction of massive flow cytometry datasets. The source code of GigaSOM.jl is available from GitHub (https://github.com/LCSB-BioCore/GigaSOM.jl) under the OSI-approved Apache-2.0 License.

Level of Interest

Please indicate how interesting you found the manuscript: Choose an item.

Quality of Written English

Please indicate the quality of language in the manuscript: Choose an item.

Declaration of Competing Interests

Please complete a declaration of competing interests, considering the following questions:

- Have you in the past five years received reimbursements, fees, funding, or salary from an organisation that may in any way gain or lose financially from the publication of this manuscript, either now or in the future?
- Do you hold any stocks or shares in an organisation that may in any way gain or lose financially from the publication of this manuscript, either now or in the future?

- Do you hold or are you currently applying for any patents relating to the content of the manuscript?
- Have you received reimbursements, fees, funding, or salary from an organization that holds or has applied for patents relating to the content of the manuscript?
- Do you have any other financial competing interests?
- Do you have any non-financial competing interests in relation to this paper?

If you can answer no to all of the above, write 'I declare that I have no competing interests' below. If your reply is yes to any, please give details below.

I declare that I have no competing interests.

I agree to the open peer review policy of the journal. I understand that my name will be included on my report to the authors and, if the manuscript is accepted for publication, my named report including any attachments I upload will be posted on the website along with the authors' responses. I agree for my report to be made available under an Open Access Creative Commons CC-BY license (http://creativecommons.org/licenses/by/4.0/). I understand that any comments which I do not wish to be included in my named report can be included as confidential comments to the editors, which will not be published.

Choose an item.

To further support our reviewers, we have joined with Publons, where you can gain additional credit to further highlight your hard work (see: https://publons.com/journal/530/gigascience). On publication of this paper, your review will be automatically added to Publons, you can then choose whether or not to claim your Publons credit. I understand this statement.

Yes Choose an item.
